# *B2M, JAK2* and *MET* in the genetic landscape of immunotolerance in lung cancer

**DOI:** 10.18632/oncotarget.26277

**Published:** 2018-11-02

**Authors:** Montse Sanchez-Cespedes

**Affiliations:** Genes and Cancer Group, Cancer Epigenetics and Biology Program (PEBC), Bellvitge Biomedical Research Institute (IDIBELL), Hospitalet de Llobregat, Barcelona, Spain

**Keywords:** PD-L1, immunotherapy, MET, JAK2, lung cancer

Avoiding host immune surveillance is a necessary step in cancer progression and, because of this, strategies that enhance the activity of the immune system against cancer cells have led to a flurry of activity in developing oncological drugs [1–2]. Similar to what happened with the development of tyrosine kinase inhibitors (TKIs), the exploitation of immune-checkpoint inhibitors (ICIs) represents a major breakthrough and a paradigm shift in cancer treatment, producing dramatic and durable responses in groups of previously incurable patients. For example, one of the immune-based strategies currently used to treat several types of malignancies, including lung cancer (LC), is the blockade of the interaction between programmed cell death-1 (PD-1) receptor, which is expressed in activated T and B cells, and its cognate ligand, PD-L1, which is expressed in the macrophage lineage and in some carcinomas. The blockade inhibits the proliferation and functions of T cells [3–6]. Despite the promise of immune-related therapeutics, they have some drawbacks, including primary resistance, such that in non-small cell lung cancer (NSCLC) only about 19–45% of unselected patients and 40–45% of those with PD-L1- positive tumors will benefit from these treatments [3, 5–6]. Furthermore, similar to what happens with TKIs and other cancer treatments, clinicians have to deal with the problem of acquired resistance to ICIs.

To take advantage of the efficacy of the currently available ICIs, and to encourage the development of novel compounds, we need to deepen our understanding of the genetic and molecular underpinnings that allow cancer cells to escape immune surveillance. In a genome-wide screening of NSCLC patient-derived xenografts (PDXs) we identified alterations of the b2-microglobulin (*B2M*) gene, which is the conserved subunit of the HLA class-I complex. We also established, in a validation cohort, that *B2M* is recurrently inactivated in around 10 percent of LCs [7]. In parallel, the same study showed that at least one-third of NSCLCs showed no detectable level of the HLA-I complex, which was correlated with low levels of PD-L1 and of tumor-infiltrating lymphocytes (TILs). Gene alterations in molecules involved in the maturation process of the HLA-I complex (e.g., *CALR, HLA-A, PDIA3,* or *TAP1*) have also been reported, although the frequency is very low [7]. These observations are evidence that, to escape immune surveillance, LC cells develop deficiencies in immune-recognition. Inasmuch as inactivation of *B2M* and other related genes is relatively infrequent, accounting for only one-third of NSCLCs lacking detectable HLA-I complex on the cell surface, we searched for additional genetic mechanisms that preclude proficient immune-surveillance in LC. In our recent study [8], we reported that the genetic activation of *MET* in NSCLC was associated with high levels of PD-L1, whereas mutations at *STK11* were associated with negative PD-L1 staining and low levels of CD8 T-lymphocyte intratumoral infiltration (TIL). Functional observations using LC cell lines confirmed that MET activation promotes the transcriptional activation of PD-L1, among other inhibitory checkpoints. The underlying reasons for this are unknown, although establishing an immunosuppressive environment may be a requirement for the oncogenic features of MET. In contrast, the use of genetically modified LC cell lines allowed us to demonstrate that STK11 neither directly regulates the levels of PD-L1 nor the appropriate expression or localization of the HLA-I complex. This is intriguing and indicates that, rather than controlling the intrinsic response, *STK11* inactivation affects the immune milieu of the tumor microenvironment.

On the other hand, IFNγ, a cytokine that is critical for innate and adaptive immunity, is an inducer of the immune response [9]. The binding of IFNγ to its cognate receptor activates JAK1 and JAK2, resulting in the transcriptional activation of large sets of genes, including some involved in antigen presentation. Secondarily, the action of IFNγ up-regulates transcripts, such as PD-L1, that inhibit the immune response as part of a broader feedback inhibition process controlling the immune response. In our work, we also explored a possible common node between the MET and the IFNγ pathways in the up-regulation of PD-L1 and found that MET-dependent up-regulation of PD-L1 is independent of IFNγ. While testing this, we discovered that some LC cell lines were refractory to the administration of IFNγ, which we found to be due to the presence of *JAK2*-inactivating mutations. Loss-of-function mutations in *JAK2* appeared concomitantly with alterations of *STK11* and *MET*, but not with genetic deficiencies at antigen presentation-related proteins.

**Figure 1:**
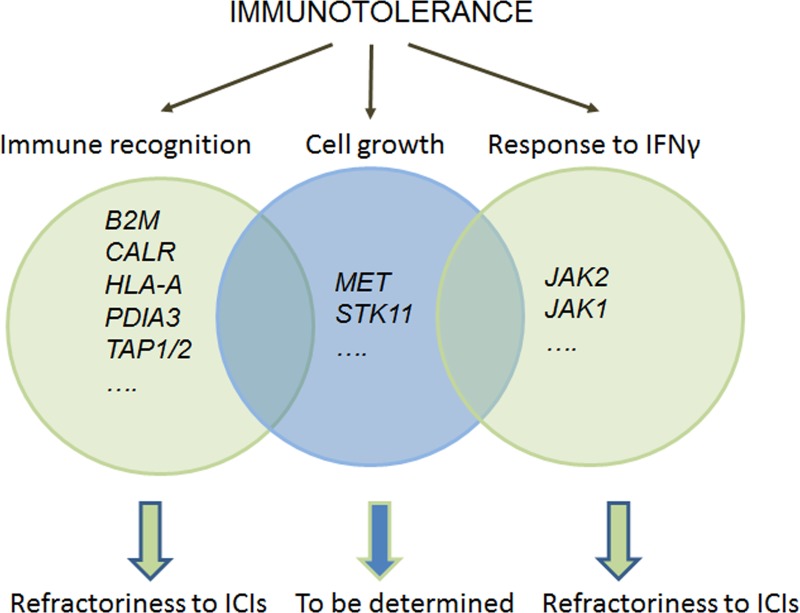
The alterations identified to date that are believed to account for immune tolerance in lung cancer can be classified into those that: a) affect genes coding for proteins directly involved in the immune response (in light green), i.e., immune recognition and response to IFNγ, or b) contain alterations in known oncogenes or tumor suppressors (in blue), whose action, for reasons that are not yet clear, involves the suppression of the immune response

Overall, we reported the presence of genetic alterations associated with proteins in lung carcinomas that are involved in the control of the immune response (Figure 1). These alterations are likely to facilitate tumor growth by allowing tumor cells to escape immune surveillance, and may also affect the response to immunotherapies. For example, loss-of-function mutations of genes coding for proteins involved in the HLA-I-complex, or of *JAK2,* may predict refractoriness to ICIs. In fact, observations in melanoma patients attest to the involvement of these gene mutations in primary and acquired resistance to these treatments [5, 10]. Conversely, lung cancers with a proficient HLA-I complex and an appropriate response to IFNγ, and that harbor activating mutations at *MET*, will restrain the immune response through the transcriptional control of immunosuppressive molecules. Lung tumors with such a genetic context may be amenable to treatment with ICIs.
